# 419. SARS-CoV-2 Environmental Surface Contamination of Healthcare Staff Common Areas

**DOI:** 10.1093/ofid/ofab466.619

**Published:** 2021-12-04

**Authors:** Helen L Zhang, Brendan Kelly, Michael Z David, Ebbing Lautenbach, Elizabeth Huang, Selamawit Bekele, Pam C Tolomeo, Emily C Reesey, Sean Loughrey, David A Pegues, Matthew J Ziegler

**Affiliations:** 1 University of Pennsylvania Perelman School of Medicine, Philadelphia, PA; 2 Hospital of the University of Pennsylvania, Philadelphia, PA; 3 University of Pennsylvania, Philadelphia, PA; 4 Perelman School of Medicine at the University of Pennsylvania, Philadelphia, PA

## Abstract

**Background:**

There are limited data regarding SARS-CoV-2 (SC2) environmental contamination in staff areas of healthcare settings. We performed environmental sampling of staff areas in wards where coronavirus disease 19 (COVID-19) patients received care and compared findings to surfaces within COVID-19 patient rooms.

**Methods:**

The study was conducted at the Hospital of the University of Pennsylvania (Philadelphia, PA) from 9/15/20-1/26/21. Sampling of 20cm^2^ surfaces in staff common areas (breakroom high-touch surfaces comprising tables and microwave/refrigerator handles; bathroom surfaces comprising toilet, sink, and doorknob; and floors), nurse workstations (computer mice and floors), and COVID-19 patient rooms (high-touch surfaces comprising bedrail, computer mice/keyboards, and doorknobs; bathroom surfaces; and floors) was performed using flocked swabs one or more times per week. Specimens underwent RNA extraction and quantitative real-time polymerase chain reaction to detect the SC2 N1 region. Median comparisons were performed using Wilcoxon rank sum test. Trends in odds were evaluated using Score test.

**Results:**

Proportions of surface specimens with detectable SC2 RNA are summarized in Table 1. Median copy numbers were lower among staff toilets compared to COVID-19 patient toilets (135.6 vs. 503.8 copies/specimen, p=0.02), lower among staff breakroom compared to patient room high-touch surfaces (104.3 vs. 220.3 copies/specimen, p=0.007), and similar between staff and patient room samples from sinks and floors. At nurse workstations, SC2 RNA was detected among 22/177 (12.4%) computer mouse and 147/178 (82.6%) floor samples. Odds of SC2 detection increased by study week among common area (p< 0.001) and nurse workstation samples (p< 0.001) (Figures 1 and 2).

Table 1. SARS-CoV-2 (SC2) RNA detection on staff common area and coronavirus disease 19 (COVID-19) patient room surfaces at the Hospital of the University of Pennsylvania, 9/15/20-1/26/21.

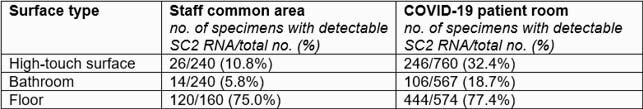

Figure 1. Proportion of environmental surface specimens with detectable SARS-CoV-2 RNA from a) staff common areas and b) nurse workstations of inpatient wards where coronavirus disease-19 patients received care at the Hospital of the University of Pennsylvania, 9/15/20-1/26/21.

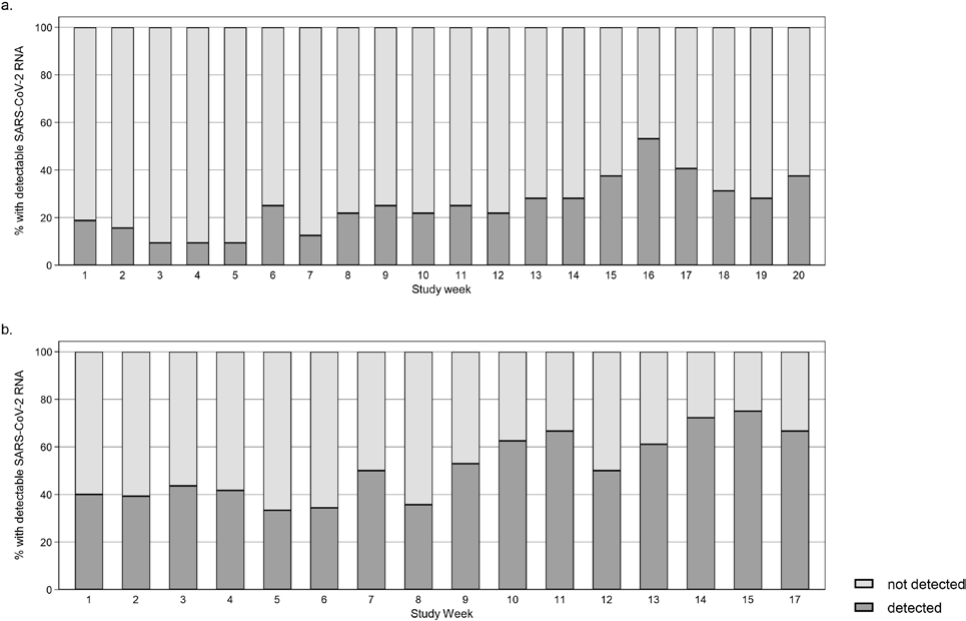

Figure 2. Proportion of environmental surface specimens with detectable SARS-CoV-2 RNA in staff common areas of inpatient wards where coronavirus disease-19 patients received care at the Hospital of the University of Pennsylvania, 9/15/20-1/26/21, by surface type: a) staff breakroom surfaces, b) staff bathroom surfaces, c) staff common area floors.

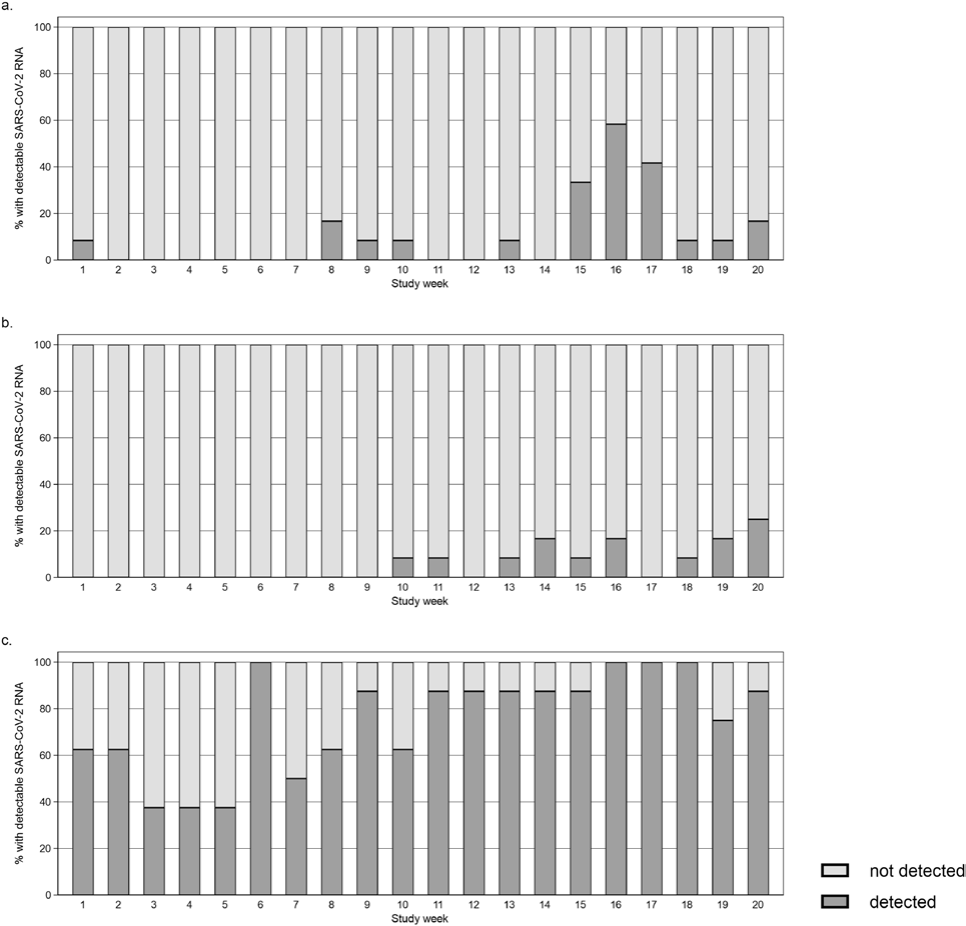

**Conclusion:**

A low prevalence of detectable SC2 RNA was observed among staff area high-touch surfaces; however, the likelihood of detection increased over time. Environmental SC2 RNA detection may reflect primary contamination from infected healthcare workers or secondary contamination from contact with infected patients, though a direct relationship between surface SC2 RNA viral detection and transmission risk has not been established.

**Disclosures:**

**Michael Z. David, MD PhD**, **GSK** (Board Member) **Ebbing Lautenbach, MD, MPH, MSCE**, **Merck** (Other Financial or Material Support, Member of Data and Safety Monitoring Board (DSMB))

